# A Novel Approach to Non-Destructive Rubber Vulcanization Monitoring by the Transient Radar Method

**DOI:** 10.3390/s22135010

**Published:** 2022-07-02

**Authors:** Salar Tayebi, Ali Pourkazemi, Nicolas Ospitia Patino, Kato Thibaut, Olsi Kamami, Johan Stiens

**Affiliations:** 1 Department of Electronics and Informatics, Vrije Universiteit Brussel, 1050 Brussels, Belgium; apourkaz@etrovub.be (A.P.); nicolas.ospitia.patino@vub.be (N.O.P.); kato.thibaut@vub.be (K.T.); olsi.kamami@vub.be (O.K.); jstiens@etrovub.be (J.S.); 2 Department of Mechanical Engineering, Vrije Universiteit Brussel, 1050 Brussels, Belgium

**Keywords:** vulcanization monitoring, curing time, TRM, non-destructive, complex permittivity

## Abstract

Rubber is one of the most used materials in the world; however, raw rubber shows a relatively very low mechanical strength. Therefore, it needs to be cured before its ultimate applicatios. Curing process specifications, such as the curing time and temperature, influence the material properties of the final cured product. The transient radar method (TRM) is introduced as an alternative for vulcanization monitoring in this study. Three polyurethane-rubber samples with different curing times of 2, 4, and 5.5 min were studied by TRM to investigate the feasibility and robustness of the TRM in curing time monitoring. Additionally, the mechanical stiffness of the samples was investigated by using a unidirectional tensile test to investigate the potential correlations between curing time, dielectric permittivity, and stiffness. According to the results, the complex permittivity and stiffness of the samples with 2, 4, and 5.5 min of curing time was 17.33 ± 0.07 − (2.41 ± 0.04)j; 17.09 ± 0.05 − (4.90 ± 0.03)j; 23.60 ± 0.05 − (14.06 ± 0.06)j; and 0.29, 0.35, and 0.38 kPa, respectively. Further statistical analyses showed a correlation coefficient of 0.99 (*p* = 0.06), 0.80 (*p* = 0.40), and 0.92 (*p* = 0.25) between curing time–stiffness, curing time–permittivity (real part), and curing time–permittivity (imaginary part), respectively. The correlation coefficient between curing time and permittivity can show the potential of the TRM system in contact-free vulcanization monitoring, as the impact of vulcanization can be tracked by means of TRM.

## 1. Introduction

**Figure 1:**
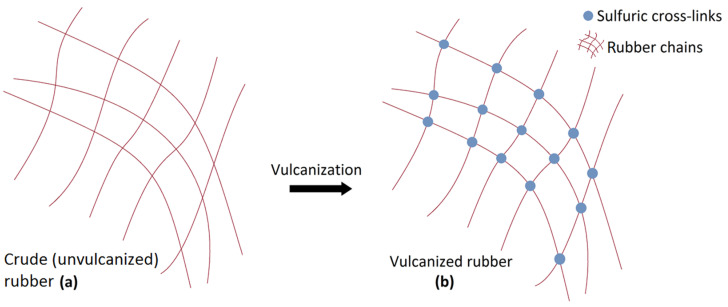
Schematic of the sulfur vulcanization process. (**a**) Crude (unvulcanized) rubber. (**b**) Vulcanized rubber. By forming cross-links between different sections of the polymer chain, a strong three-dimensional molecular network will be formed to increase the strength of rubber.

Rubber is one of the most used materials in the world. In contrast to other engineering materials, it can provide strong attributes and highly deformable characteristics at the same time [1,2,3]. Its unique features make it an important product to be used in diverse fields ranging from flexible tubing or absorbing system manufacturing for automobile, construction, agricultural, and aerospace industries to sealing and packaging applications in healthcare, petroleum, food, and beverage industries [4,5,6]. However, raw rubber is a relatively soft material that shows very poor mechanical strength. Consequently, it needs to be cured before it becomes suitable to roll it out in various applications. This process is known as vulcanization, a range of processes for hardening rubbers that plays an important role in the final characteristics of rubber [7]. It works by forming cross-links between different sections of the polymer chain, leading to a strong three-dimensional molecular network [7,8]. Ultimately, it increases the stiffness and durability of the rubber in addition to other changes in its mechanical and electromagnetic characteristics [7,8]. There are different reported curing systems to be used for rubber vulcanization, including a sulfur, dicumyl peroxide/coagent, and radiation/coagent vulcanization system [9]. Among the mentioned methods, sulfur curing systems are the most widely used vulcanization systems for rubber compounds, forming sulfuric cross-links between the rubber chains (see Figure 1) [10]. It is obvious that the curing process specifications, such as the curing time and temperature, influence the mechanical as well as electromagnetic properties of the final cured product [8]. Therefore, the optimum curing time determination at a certain curing temperature is of great importance to ensure the high performance of the final product.

Different techniques for measuring the impact of the curing time have been reported in literature [10,11,12,13,14]. For instance, differential scanning calorimetry (DSC), mass swelling analysis, tensile test, terahertz waves spectroscopy, porosity investigation, ultrasound imaging, attenuated total reflection (ATR), and Fourier-transform infrared (FTIR) spectroscopy are the frequently used assessment techniques of the vulcanization process. However, most of the reported evaluation techniques are destructive. Additionally, some evaluation techniques, including DSC, porosity, and shear stress determination, are not accurate, and the final error value might be too high (around 20%) [11]. Evaluation time is another important parameter to be considered for the vulcanization assessment techniques. For instance, mass swelling analysis needs approximately 72 h for its measurement procedure, which is relatively longer compared to other measurement methods. Although some analysis techniques like terahertz wave and ATR-FTIR spectroscopy are promising to be used for vulcanization monitoring, they are not able to deal with thick samples, as their penetration depth is limited (a few μm of penetration depth) [14,15]. In other words, rubber characterization on the basis of terahertz wave and ATR-FTIR spectroscopy might have high error, since the tests are done according to the surface and sub-surface characteristics, and no information from deeper regions can be obtained. An overview of the conventional vulcanization monitoring techniques is presented in Table 1.

Moreover, most of the conventional vulcanization monitoring techniques deal with mechanical characterization of the rubber and do not consider its electromagnetic characteristics; however, for several industries (e.g., radar absorbing materials and stealth technology), it is important to have an accurate estimation of the electromagnetic properties as well. Although there are some techniques for the electromagnetic characterization of the rubber (as a function of curing time) [10,13], their complex measurement set-up limits their usability. Therefore, the development of a novel evaluation technique that is also capable of determining the electromagnetic properties of rubber as a function of curing time could be necessary.

In the first part of this article, a new evaluation method for the curing degree of rubber on the basis of the complex permittivity calculation is presented. The geometric and electromagnetic characteristics of three polyurethane-rubber samples with different curing times were investigated by means of a fully blind, contact-free technique, known as the transient radar method (TRM) [16]. In the second part of this study, however, stiffness of the samples has been investigated by means of a unidirectional tensile test. Subsequently, statistical analyses have been performed between curing time, permittivity (real and imaginary part), and stiffness of the samples to investigate the potential correlation between them. The aim of this research was to investigate the robustness of the TRM system in finding the optimum curing time based on the electromagnetic characteristics of rubber samples. This investigation would also promote research in this field, as it is a completely novel approach to vulcanization monitoring.

## 2. Materials and Methods

### 2.1. TRM System

**Figure 2:**
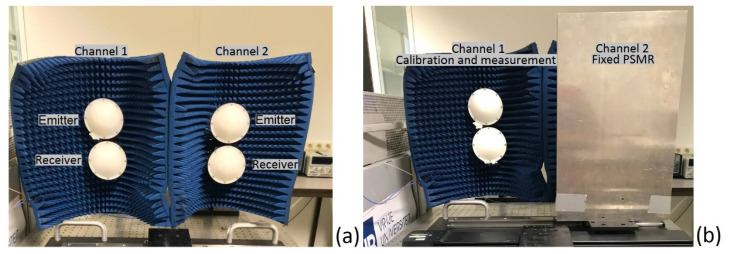
Dual channel bi-static radar system of the transient radar method (TRM). (**a**) The emitter and receiver antenna in channel 1 and 2. (**b**) Channel 1 is used for calibration and sample measurement, while channel 2 radiates towards a fixed perfect smooth metallic reflector (PSMR) for drift compensation.

The TRM measurement set-up is in fact a dual-channel bi-static radar system, which includes an emitter and receiver antenna at each channel. As shown in Figure 2, channel 1 is for calibration and sample measurement, while channel two radiates towards a fixed perfect smooth metallic reflector (PSMR) to modify drift during measurements. 

**Figure 3:**
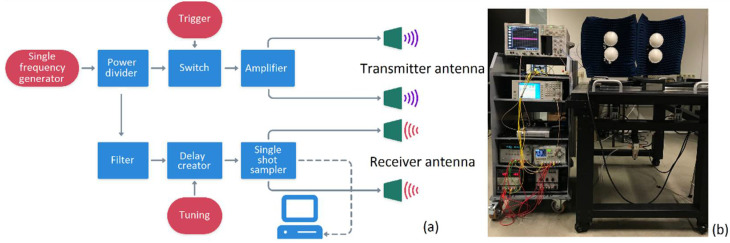
Transient radar system in detail. (**a**) A block diagram showing different TRM modules. (**b**) Different components of the TRM system.

In addition to the illumination channels, there are several other components that run the TRM system (see Figure 3). The first module is the single frequency generator, which is a voltage-controlled oscillator (VCO) that generates a continuous electromagnetic wave in single note. The power divider has been used as the next module in order to split the output of the single frequency generator into two similar (power) amplitudes and phases for each channel of the differential set-up. The reflective single-pole-single-throw (SPST, switches with one input and only one output terminal) switch is the next module that has two main functions. Rise time generation by means of a single harmonic and rise time modulation by a frequency carrier shift from the baseband to intermediate band is its first task, while the second task is to reflect the signal at the toggling moment from the conductive to non-conductive condition in order to trigger the single shot sampler. To increase the signal-to-noise ratio (SNR) for further signal processing, an amplifier has been used in the system to increase the amplitude of the signal radiated towards the testing sample. The single shot sampler is the next module that records the amplitude of the reflected signal in an infinitesimal time interval (femtosecond). To help the operator to record the reflected waves at a certain timeframe, a delay creator was used in the system as well. The trigger is the last module that sends the commands to the SPST switch for toggling from the non-conductive to conductive condition or vice versa [16]. In this investigation, an excitation signal with an input power of 100 mW (1 ns rise time and 4 ns of total exposure) has been used for the TRM system; however, higher input powers might be deployed to have an increased penetration depth for certain materials (e.g., construction structures). Temperature, humidity, and distance are the other important parameters to be considered for TRM measurements. This investigation was done at the lab environment with a temperature of 24 °C. To minimize the impact of environmental factors, the temperature and humidity were kept constant during the measurements. Concerning the distance, all the measurements were done at the radiating near field (Fresnel region). According to the frequency (10 GHz) and the antenna aperture (10 cm), the measurements were performed at 20 cm from the front side of the rubber samples.

**Figure 4:**
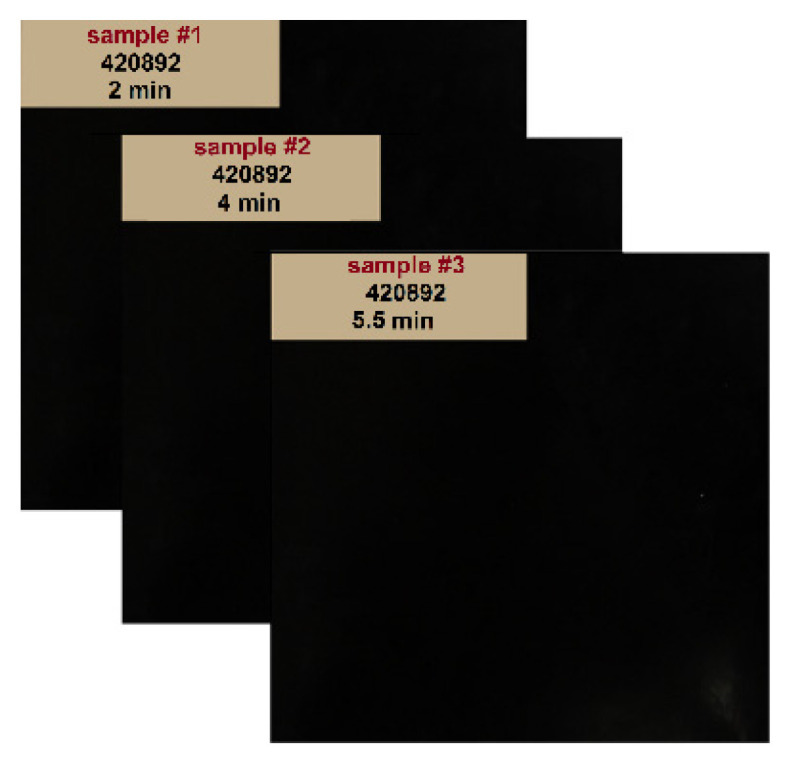
Rubber samples with different curing times of 2, 4, and 5.5 min.

Three polyurethane-rubber samples (30 cm × 30 cm) with different curing times of 2, 4, and 5.5 min were provided by a third party for further investigations by means of TRM (see Figure 4). Subsequently, each rubber sample was attached to a rigid frame to ensure their vertical position with respect to the emitter and receiver antennas. Strong absorber sheets were also placed behind the samples to mitigate the environmental interference. After the TRM measurements and before the tensile test, the thickness of each sample was measured by a thickness gauge with an accuracy of 0.03 mm (each measurement was repeated 5 times at different locations of the sample). 

### 2.2. Electromagnetic and Geometric Properties Extraction

**Figure 5:**
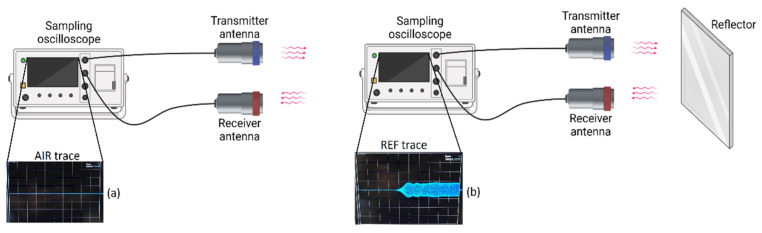
Two-step calibration process. (**a**) AIR trace when there is no object in front of the antennas. Consequently, the crosstalk between the antennas can be recorded. (**b**) REF trace when there is a PSMR in front of channel 1.

The two-step calibration of the TRM set-up was the first step to be done before the main measurements (see Figure 5). At first, the crosstalk between the antennas was measured when no object was in channel 1. This signal is known as the “AIR” signal, as air is the only dielectric in front of the antennas. In the second step, reflection signal was measured when a PSMR was in front of the transmitter antenna in channel 1. This signal is known as the “REF” signal, since the reflection is from a reflector. Subsequently, TRM calibration [17] was done by knowing the upper and lower reflection extremities. 

**Figure 6:**
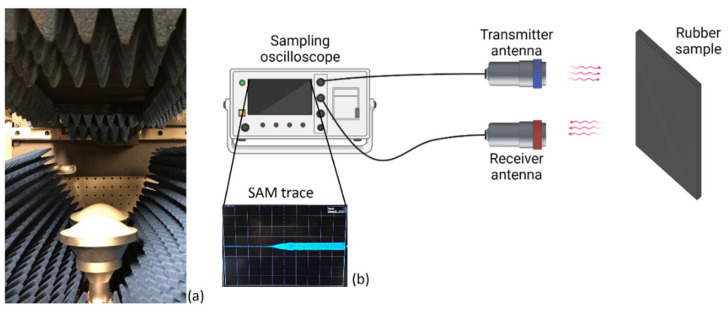
Sample measurement by TRM. (**a**) The rubber sample in front of the transmitter and receiver antennas. (**b**) A representation of the SAM trace obtained from the sample under investigation.

After the calibration procedure, the measurement of each rubber sample was started; the 10-GHz transient-radar signal was radiated towards the rubber samples (see Figure 6), and its time-dependent reflection was recorded. This signal was named “SAM”, as the reflection was from the sample under investigation.

Using the histogram technique, the recorded (raw) signal was converted to the smooth one [16]. In this technique, the areas with the highest density of sample points in amplitude-time plane were determined by a density-weighted averaging method. To minimize the impact of noise, jitter, and drift, the averaging process was restricted to 10 measurements in order to have the least difference between the repeated measurements. Subsequently, drift was calculated in channel 2 and mitigated from the signals obtained from channel 1. As the next step, the “AIR” signal was subtracted from the “SAM” and “REF” signals to remove the crosstalk between the antennas. The antennas used in this experience had a normal gain; thus, diffraction from the output apertures was relatively large. Therefore, there was a large crosstalk between antennas once the angle of antenna’s orientations was less than 5 degrees. However, in this condition, we could use the assumption of perpendicular illumination for the signal processing in later stages. After removing the crosstalk, the “nose” of the signal, defined as the shortest round-trip-time (RTT) between the antenna and the front side of the rubber sample, was determined by reconciling the “AIR” and “REF” signals and finding their earliest intersection (for better understanding. 

**Figure 7:**
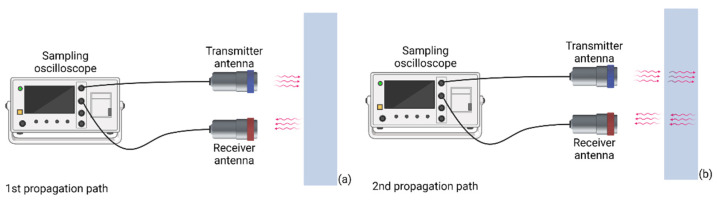
A schematic of the propagation paths in a single-layer structure. (**a**) First propagation path between the antennas and the front side of the sample. (**b**) Second propagation path that also penetrates through the sample under test.

To extract the electromagnetic and geometric properties of the rubber samples, we had to decompose the reflection signal (when the crosstalk was removed, “SAM-AIR”) into different propagation paths (pp). In fact, each pp represents a possibility for the electromagnetic waves to propagate through the sample under test (SUT). Considering this definition, the first pp includes the shortest RTT between the transmitter antenna and the front side of the sample. The second pp, however, represents the wave radiation from the transmitter antenna towards the sample under test, transmission through the first interface, penetration inside the sample, reflection from the backside of the sample, and transmission through the first interface and towards the receiver antenna (see Figure 7). Since the rubber samples used in this investigation had only one layer, detecting the first and second pp would be enough. 

Propagation paths can be obtained by trial and error to rebuild the initial part of each “SAM-AIR” signal based on the “REF-AIR” signal. Therefore, the amplitude and phase changes applied to the “REF-AIR” signal, to find the initial part of the “SAM-AIR” signal, could be obtained. Taking all the explanations into account, the first propagation path can mathematically be represented as:

(1) $$PP_{\left(t0\right)}=A\Gamma_{01}e^{-j2\beta_{0}d_{0}}e^{j\omega_{0}t}U(t-t_{0}),t>t_{0}\;$$

where *A,* Γ_01_*, β*_0_*, d*_0_*, ω*_0_*, t, t*_0_*, U(t),* and *PP*_(*t*0)_ refer to the amplitude, reflection coefficient from the front side, propagation constant in free space, the distance between antennas and SUT, angular frequency, time, round trip time between the antennas and SUT, Heaviside function, and first propagation path, respectively. In a similar way, the second propagation path can be written as:

(2) $$PP_{\left(t_{0}+t_{1}\right)}=(\frac{PP_{\left(t_{0}\right)}}{\Gamma_{01}})T_{01}T_{10}e^{-2\alpha_{1}d_{1}}e^{-2j(\beta_{0}d_{0}+\beta_{1}d_{1})}\Gamma_{10}e^{j\omega_{0}t}U(t-t_{0}-t_{1}),t>t_{0}+t_{1}$$

where *T*_01_, *T*_10_, *β*_1_, *d*_1_, *t*_1_, and α_1_ refer to the transmission coefficient through the first interface, transmission coefficient through the second interface, propagation constant in the material, thickness of SUT, RTT, and attenuation coefficient in sample, respectively.

Having the propagation paths, one can calculate the time delay between the first and second propagation paths. This time delay can be converted to the sample thickness, as the time delay is a function of the distance and material-dependent speed of light. In general, using the TRM system, we can monitor the curing time based on the reflection response of each sample within a couple of minutes. However, to extract all the geometric and electromagnetic properties of the samples, 2–3 h might be needed, depending on the processing power of the hardware, type of the sample, thickness, etc.

### 2.3. Unidirectional Tensile Test

**Figure 8:**
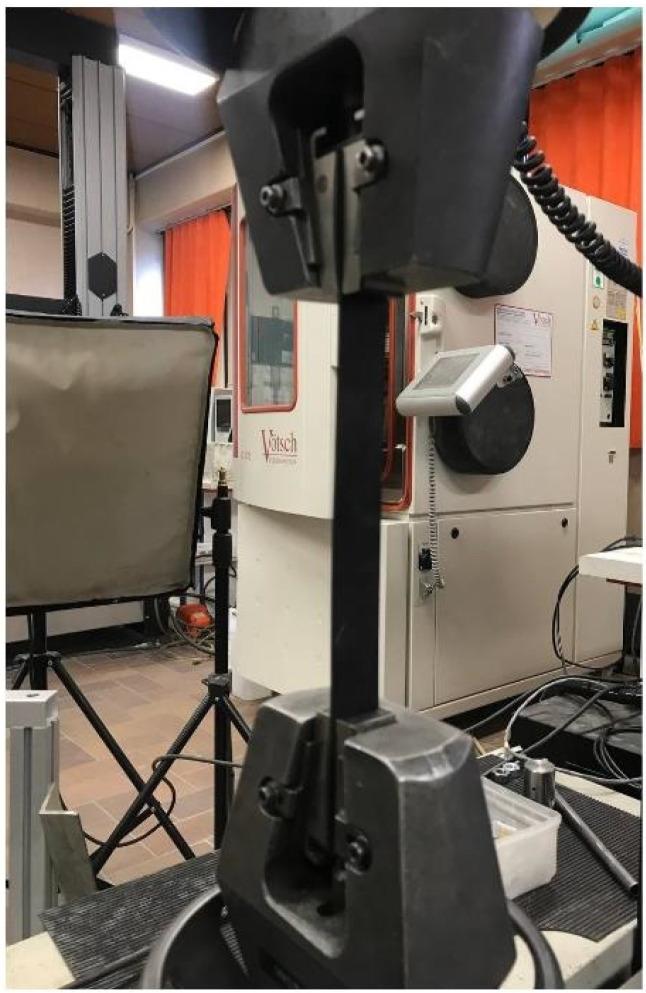
Unidirectional tensile test performed on rubber samples with different curing times.

Following the electromagnetic characterization of the rubber samples, a unidirectional tensile test was used to investigate the stiffness of the samples versus curing time (see Figure 8). Taking the ASTM D412 guidelines into account, each rubber sample with the curing time of 2, 4, and 5.5 min was cut into 2.5 cm × 30 cm pieces (straight geometry for each sample). The tensile tests were done using an Instron universal testing system (Instron Inc., corporation, MA, USA) at 23 °C. Because of the very high elasticity of the samples and measurement limitations, the elongation applied to each sample was restricted to 30 cm. Subsequently, an elongation of 30 cm with the stretch rate of 20 mm/min was applied to the samples. Each rubber sample was tested three times. Subsequently, the force–displacement graphs of each sample were obtained. Having the initial length and the cross-sectional area of each sample, the force–displacement graphs were converted into the stress–strain graphs.

### 2.4. Statistical Analyses

After obtaining the electromagnetic and mechanical characteristics of the rubber samples, a Pearson’s correlation analysis was done by MATLAB (MathWorks, Inc., Natick, MA, USA). A dataset including real and imaginary values of permittivity, speed of light, curing time, and stiffness was generated. Subsequently, the correlation study was done between each pair of variables to determine the correlation coefficient. Since the number of samples (with different curing time) was limited, no threshold for the *p*-value was defined. However, the obtained *p*-value is reported to provide a thorough overview of this investigation.

## 3. Results

### 3.1. Geometric and Electromagnetic Properties

**Figure 9:**
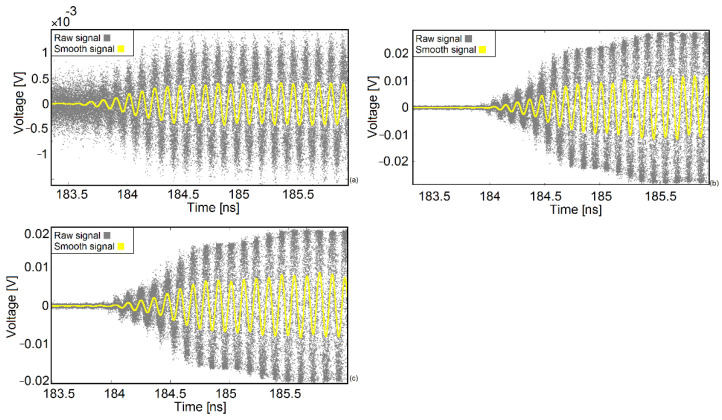
Raw and smooth signals of rubber samples. (**a**) Sample after 2 min of vulcanization. (**b**) Sample after 4 min of vulcanization. (**c**) Sample after 5.5 min of vulcanization.

After the calibration process, the SAM trace was recorded using the single shot sampler. The raw data of each rubber sample are presented in Figure 9. Subsequently, the raw data were converted into the smooth signals by means of the histogram technique. 

**Figure 10:**
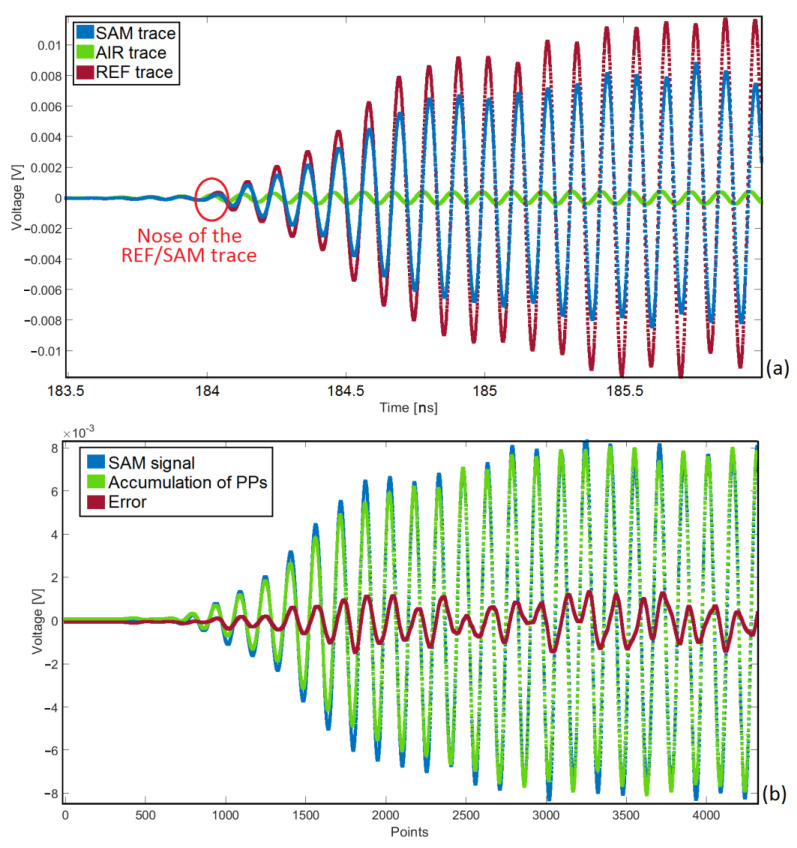
Last step of TRM signal processing to obtain the propagation paths (PPs). (**a**) REF, AIR, and SAM trace. By reconciling the REF/SAM and AIR traces, a certain moment could be found that the REF/SAM trace started to deviate from the AIR trace. This is the nose of the signal, the certain moment that the reflections from the first interface (frontside of the SUT) will be detected. (**b**) Accumulation of PPs to re-generate the “SAM-AIR” signal. Error is illustrated as well.

After obtaining the smooth signals, further signal processing was done to remove the drift, switch leakage, offset, etc. Finally, the “REF-AIR” signal was used to re-generate the “SAM-AIR” signal based on the accumulation of the propagation paths (see Figure 10). Having the propagation paths, the geometric and electromagnetic properties of each rubber sample were obtained (see Table 2).

As shown in Table 2, the thickness (measured with a thickness gauge) of the rubber samples with 2, 4, and 5.5 min of curing time was 2.41 ± 0.11, 2.21 ± 0.07, and 2.18 ± 0.15 mm, respectively. Using the TRM system, the thickness of each sample was 2.47 ± 0.11, 2.27 ± 0.17, and 2.36 ± 0.21 mm, determined with a relative error of 2.48%, 2.71%, and 3.49%, respectively. 

The complex permittivity of the samples was obtained as 17.33 ± 0.07 − (2.41 ± 0.04)j, 17.09 ± 0.05 − (4.90 ± 0.03)j, and 23.60 ± 0.05 − (14.06 ± 0.06)j. Although the imaginary part of the permittivity increased by increasing the curing time from 2 to 4 min, the real part did not change significantly in this interval. However, when the curing time increased from 4 to 5.5 min, a significant elevation in the real and imaginary parts of permittivity could be observed.

Having the complex permittivity of each sample, we calculated the speed of light through each rubber sample. The speed of light in every medium (once the illumination is perpendicular) can be calculated using the following equation [15]. Although the calculation of speed of light once the illumination is oblique is more complex, using Equation (3) is a good approximation in determining the speed of light in the rubber samples.

(3) $$V=\frac{\sqrt{2}C}{\sqrt{\mu^{'}\varepsilon^{'}-\mu^{"}\varepsilon^{"}+\sqrt{{\left(\mu^{'}\varepsilon^{'}-\mu^{"}\varepsilon^{"}\right)}^{2}+{\left(\mu^{"}\varepsilon^{'}-\mu^{'}\varepsilon^{"}\right)}^{2}}}}$$

Since the samples used in this study were non-magnetic, we can re-write the speed of light equation as:

(4) $$V=\frac{\sqrt{2}C}{\sqrt{\varepsilon^{'}+\sqrt{\varepsilon^{'}{}^{2}+\varepsilon^{"}{}^{2}}}}$$

According to Equations (3) and (4), the speed of light in rubber samples with 2, 4, and 5.5 min of vulcanization was 7.1842 × 10^7^, 7.1799 × 10^7^, and 5.9327 × 10^7^ ms^−1^, respectively.

### 3.2. Mechanical Properties

**Figure 11:**
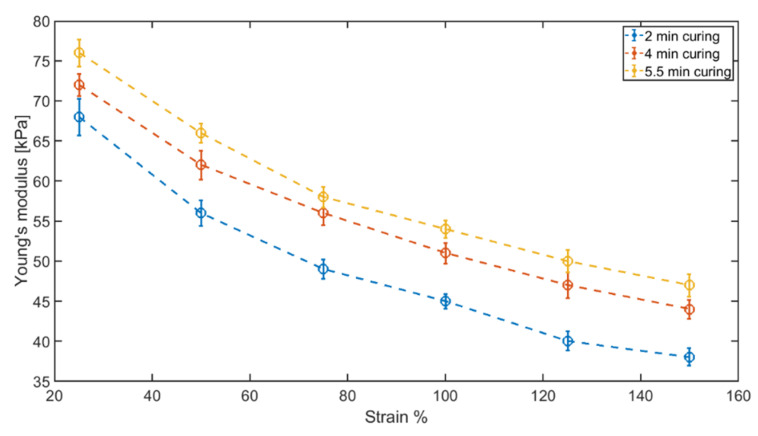
Young’s modulus of each rubber sample. The Young’s modulus of each sample was measured at 25%, 50%, 75%, 100%, 125%, and 150% strain values.

Following the electromagnetic properties extraction of the rubber samples, the unidirectional tensile test was done on the samples. The stiffness of the rubbers versus strain is provided in Figure 11. In this study, the stiffness of each rubber sample is reported based on the stress–strain curves at 25%, 50%, 75%, 100%, 125%, and 150% strain. Young’s modulus of each rubber sample at different strain percentages are shown in Table 3.

### 3.3. Statistical Analyses

**Figure 12:**
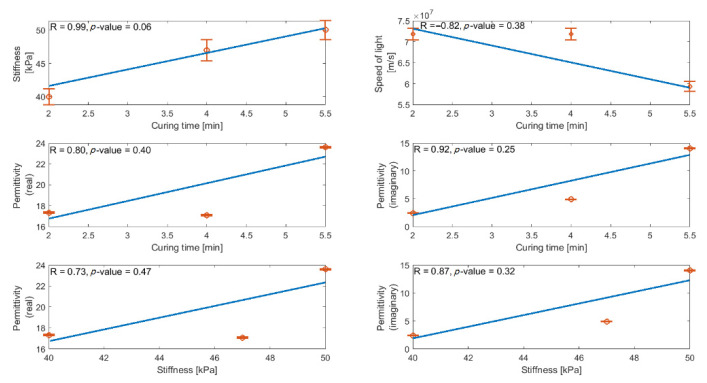
Correlation matrix obtained by means of Pearson’s correlation analysis between curing time, stiffness, real, and imaginary parts of permittivity.

To measure the strength of the linear relationship between curing time, permittivity, and stiffness, Pearson’s correlation test was done between the obtained results in previous sections. A dataset consisting of curing time, stiffness, speed of light, real part of permittivity, and imaginary part of permittivity was defined in MATLAB. Subsequently, the correlation test was done between each pair of the variables. The correlation coefficient and *p*-value were calculated as well (Figure 12).

The strongest correlation was observed between curing time–stiffness (R = 0.99, *p* = 0.06). A relatively high correlation was also observed between curing time and the real and imaginary parts of the permittivity (R = 0.80, *p* = 0.40 and R = 0.92, *p* = 0.25, respectively). Since only three samples (with different curing time) were available in this study, the obtained *p*-value was not significant. 

## 4. Summary and Discussion

The TRM system, as a contact-free method to extract the electromagnetic and geometric properties of the samples under investigation, has been introduced as a non-destructive alternative for vulcanization monitoring in this study. Three rubber samples with different curing times (2, 4, and 5.5 min) were studied by TRM to investigate the feasibility and robustness of the TRM in curing time monitoring. Additionally, the mechanical stiffness of the samples was investigated by using a unidirectional tensile test. Statistical investigations were done between curing time and electromagnetic and mechanical properties of the samples in order to investigate the potential correlations between curing time, permittivity, and stiffness.

The thicknessed of the samples with 2, 4, and 5.5 min of curing time obtained by a thickness gauge were 2.41 ± 0.11, 2.21 ± 0.07, and 2.18 ± 0.15, respectively. The same parameters obtained by TRM were 2.47 ± 0.11, 2.27 ± 0.17, and 2.36 ± 0.21, which resulted in the relative error of 2.48%, 2.71%, and 3.49%, respectively. Since TRM averages over the full area of each sample, a relatively high precision was observed by this technique. Moreover, we observed that the thickness obtained by TRM yielded larger systematic values compared with the mechanically measured ones. These larger values are most likely related to a small beam displacement when the electromagnetic beam propagates back and forth inside the sample. This should be considered as a systematic error [18]. 

The complex permittivity of the samples with 2, 4, and 5.5 min of curing time was 17.33 ± 0.07 − (2.41 ± 0.04)j, 17.09 ± 0.05 − (4.90 ± 0.03)j, and 23.60 ± 0.05 − (14.06 ± 0.06)j, respectively. Based on the principles of the TRM method, we may state that the errors we obtained are an order of magnitude smaller than for the thickness measurements, approximately. The stiffness of each sample was calculated for different levels of strain, which is reported in Table 3. A further statistical analysis showed a correlation coefficient of 0.99, 0.80, and 0.92 between curing time–stiffness, curing time–permittivity (real part), and curing time–permittivity (imaginary part), respectively. The correlation coefficient between curing time and permittivity can show the potential of the TRM system in vulcanization monitoring. 

## 5. Conclusions

Compared with the conventional rubber vulcanization monitoring techniques, including DSC, mass swelling analysis, compression set test, relaxation method, and shear stress evaluation, TRM has a significantly smaller measurement duration (a few minutes). Moreover, because of the used frequency (10 GHz) in this system, there would be no issue regarding the penetration depth, and relatively thick samples can be studied as well. The small error level is another advantage of this method in comparison with the conventional techniques, which have high error in the results. Lastly, the measurement duration of this technique is relatively short compared with other techniques. In order to check the degree of curing qualitatively, the measurement takes a few minutes, approximately. However, it may take longer, up to a couple of hours, to have the numerical values, which is longer than some of the conventional techniques but is still short enough to be used for online quality monitoring. According to this investigation with a limited number of samples, TRM can be considered as a potential non-destructive testing technique to cope with the fully blind, real-time, and contact-free vulcanization monitoring of different samples. However, we should notice that the number of samples with different curing times was limited in this investigation. Therefore, further investigation with a wider range of the curing time and higher number of samples should be performed in the future.

## Figures and Tables

**Table 1:** Conventional curing monitoring techniques adapted from [11].

Method	Destructive	Specific Geometry Needs	Error	Duration
Differential Scanning Calorimetry	Yes	None	High	<1 h
Mass swelling	Yes	None	Low	≈72 h
Tensile test	Yes	Yes	Low	<1 h
Compression set test	Yes	None	Low	≈72 h
Relaxation	Yes	Yes	Low	≈1 day
Hardness	Yes	None	Low	<1 h
Shear stress	Yes	None	High	<1 h
Porosity	Yes	None	High	<1 h
THz spectroscopy	No	None	Medium	<1 h
ATR-FTIR spectroscopy	No	Yes	Medium	<1 h

**Table 2:** The geometric and electromagnetic characteristics of the rubber samples obtained via TRM.

Curing time (min)	2.0	4.0	5.5
Thickness [mm] (caliper)	2.41 ± 0.11	2.21 ± 0.07	2.18 ± 0.15
Thickness [mm] (TRM)	2.47 ± 0.11	2.27 ± 0.17	2.36 ± 0.21
Complex permittivity	17.33 ± 0.07 − (2.41 ± 0.04)j	17.09 ± 0.05 − (4.90 ± 0.03)j	23.60 ± 0.05 − (14.06 ± 0.06)j
Relative thickness error [%]	2.48	2.71	3.49

**Table 3:** Young’s modulus of rubber samples at different strain levels.

Curing Time [min]	Young’s Modulus 25% [kPa]	Young’s Modulus 50% [kPa]	Young’s Modulus 75% [kPa]	Young’s Modulus 100% [kPa]	Young’s Modulus 125% [kPa]	Young’s Modulus 150% [kPa]
2	68 ± 2.3	56 ± 1.6	49 ± 1.2	45 ± 0.9	40 ± 1.2	38 ± 1.1
4	72 ± 1.4	62 ± 1.8	56 ± 1.5	51 ± 1.3	47 ± 1.6	44 ± 1.2
5.5	76 ± 1.7	66 ± 1.2	58 ± 1.3	54 ± 1.1	50 ± 1.4	47 ± 1.4

## Data Availability

Derived data supporting the findings of this study in addition to the processing algorithms are available from the corresponding author on request.

## References

[1] Vergnaud J.M., Rosca I.D. (2008). Rubber Curing and Properties.

[2] Hernández M., Ezquerra A.T., Verdejo R., López-Manchado A.M. (2012). Role of Vulcanizing Additives on the Segmental Dynamics of Natural Rubber. Macromolecules.

[3] Kalia S., Avérous L. (2011). Biopolymers.

[4] Al-Sabaeei A.M., Yussof N.I., Napiah M.B., Sutanto M.H. (2019). A review of using natural rubber in the modification of bitumen and asphalt mixtures used for road constuctions. J. Teknol..

[5] Borges A.F., Filho E., Miranda M.C.R., Santos M.L.D., Herculano R.D., Guastaldi A.C. (2015). Natural rubber latex coated with calcium phosphate for biomedical application. J. Biomater. Sci. Polym. Ed..

[6] Rezaeian I., Zahedi P., Rezaeian A. (2012). Rubber Adhesion to Different Substrates and Its Importance in Industrial Applications: A Review. J. Adhes. Sci. Technol..

[7] Mark J.E., Erman B. (2005). Science and Technology of Rubber.

[8] Ghoreishy M.H.R. (2015). A state-of-the-art review on the mathematical modeling and computer simulation of rubber vulcanization process. Iran. Polym. J..

[9] El-Nemr K.F. (2011). Effect of different curing systems on the mechanical and physico-chemical properties of acrylonitrile butadiene rubber vulcanizates. Mater. Des..

[10] Chueangchayaphan N., Nithi-Uthai N., Techakittiroj K., Manuspiya H. (2018). Evaluation of dielectric cure monitoring for in situ measurement of natural rubber vulcanization. Adv. Polym. Technol..

[11] Zaimova D., Bayraktar E., Dishovsky N.T. (2011). State of cure evaluation by different experimental methods in thick rubber parts. J. Achiev. Mater. Manuf. Eng..

[12] Rajaraian R. (2017). Determination of Optimum Cure State of Elastomers. https://pure.mpg.de/rest/items/item_2492336/component/file_2492335/content.

[13] Jaunich M., Stark W., Hoster B. (2009). Monitoring the vulcanization of elastomers: Comparison of curemeter and ultrasonic online control. Polym. Test..

[14] Muroga S., Takahashi Y., Hikima Y., Ata S., Ohshima M., Okazaki T., Hata K. (2021). New evaluation method for the curing degree of rubber and its nanocomposites using ATR-FTIR spectroscopy. Polym. Test.

[15] Hirakawa Y., Yasumoto Y., Gondo T., Sone R., Morichika T., Minato T., Hojo M. (2020). Application of Terahertz Spectroscopy to Rubber Products: Evaluation of Vulcanization and Silica Macro Dispersion. Electronics.

[16] Pourkazemi A., Stiens J., Becquaert M., Vandewal M. (2017). Transient Radar Method: Novel Illumination and Blind Electromagnetic/Geometrical Parameter Extraction Technique for Multilayer Structures. IEEE Trans. Microw. Theory Tech..

[17] Pourkazemi A., Tayebi S., Stiens J. (2021). Fully Blind Electromagnetic Characterization of Deep Sub-Wavelength (λ/100) Dielectric Slabs With Low Bandwidth Differential Transient Radar Technique at 10 GHz. IEEE Trans. Microw. Theory Tech..

[18] Pourkazemi A., Tayebi S., Stiens J. (2020). Error Assessment and Mitigation Methods in Transient Radar Method. Sensors.

